# Ethnobotany in a coastal environmental protected area: shifts in plant use in two communities in southern Brazil

**DOI:** 10.1186/s13002-018-0265-0

**Published:** 2018-11-03

**Authors:** Rafaela H. Ludwinsky, Natalia Hanazaki

**Affiliations:** Department of Ecology and Zoology, Laboratory of Human Ecology and Ethnobotany, Federal University of Santa Catarina, Campus Reitor João David Ferreira Lima, Florianópolis, 88040-900 Brazil

**Keywords:** Brazilian atlantic forest, Shifts and losses in plant use, Coastal areas, Ethnobotany

## Abstract

**Background:**

We investigated local knowledge of plants in terms of plant use shifts and losses, in two coastal communities within a protected area in southern Brazil. Our hypothesis is that people’s livelihoods are associated with different ethnobotanical knowledge, and changes in these activities can reflect shifts in ethnobotanical knowledge such as stopping using some plants.

**Methods:**

We interviewed 125 inhabitants after prior informed consent, asking her/him about their socioeconomic profile and to free list the plants they know. The free lists were analyzed by frequency of cited plants. To compare averages of cited plants and age in both communities, we used the Wilcoxon test with a significance of 5%. Spearman correlation was tested with number of plants cited in the past and the interviewees’ age. Permanence and change in economic activities in each community were represented using a graph and compared through a chi-squared test with a significance of 5%. Qualitative analyses of the interviews and a field diary were used to analyze driving forces for the abandonment of used plants.

**Results:**

We identified 231 plant species that were currently used mainly for food and medicine. Despite being in a protected area, most of the cited plants were exotic and cultivated in home gardens. We do not confirm the hypothesis that changes in livelihoods are reflected in the plants used; however, qualitative analyses showed potential drivers for shifts and losses of plant use. “Environmental law” and “protected area” were the drivers most related to the abandonment of plant use.

**Conclusions:**

While recognizing the importance of the protected area to maintain local people and their traditions, we documented a shift in plant use that is mainly correlated to construction activities that disappeared from daily practices.

## Background

Coastal areas in tropical countries are rapidly changing due to tourism and urbanization [1]. The development of infrastructure and economic growth can transform coastal areas in a short period [2], and these transformations can influence local knowledge and practices, causing changes over time [2–6]. While urbanization and economic growth cause pressure on natural resources and local people, one strategy used to resist some of these changes is the creation of protected areas to conserve remnants of nature in this complex scenario [7–9]. Protected areas fall under distinct categories of protection with different restrictions of use. One of the most flexible categories is category VI by the International Union for the Conservation of Nature [10], which allows human occupation and the sustainable use of natural resources, and in these cases, the conservation and use of natural resources must be combined to accomplish the goals of the protected area.

Ethnobotany can help explain how local people perceive, use, and manage plants [11]. Because ethnobotany investigates local knowledge through the body of local traditions and practices [12], it plays a key role in advancing our comprehension of possible knowledge responses driven by changes at a given point of time [13]. We investigated local knowledge of plants in terms of plant use shifts and losses, in two coastal communities within a protected area in southern Brazil. In doing so, we evaluate changes in economic activities as an influence on plant knowledge dynamics and discuss potential drivers of shifts in plant use that were mentioned by residents. Our hypothesis is that people’s livelihoods are associated with different ethnobotanical knowledge, and changes in these activities can reflect shifts such as stopping using some plants. Our prediction is that in the communities with the highest proportion of change in livelihoods, there will be a higher frequency of citations of plants used in the past.

## Methods

### Study area

**Fig. 1:**
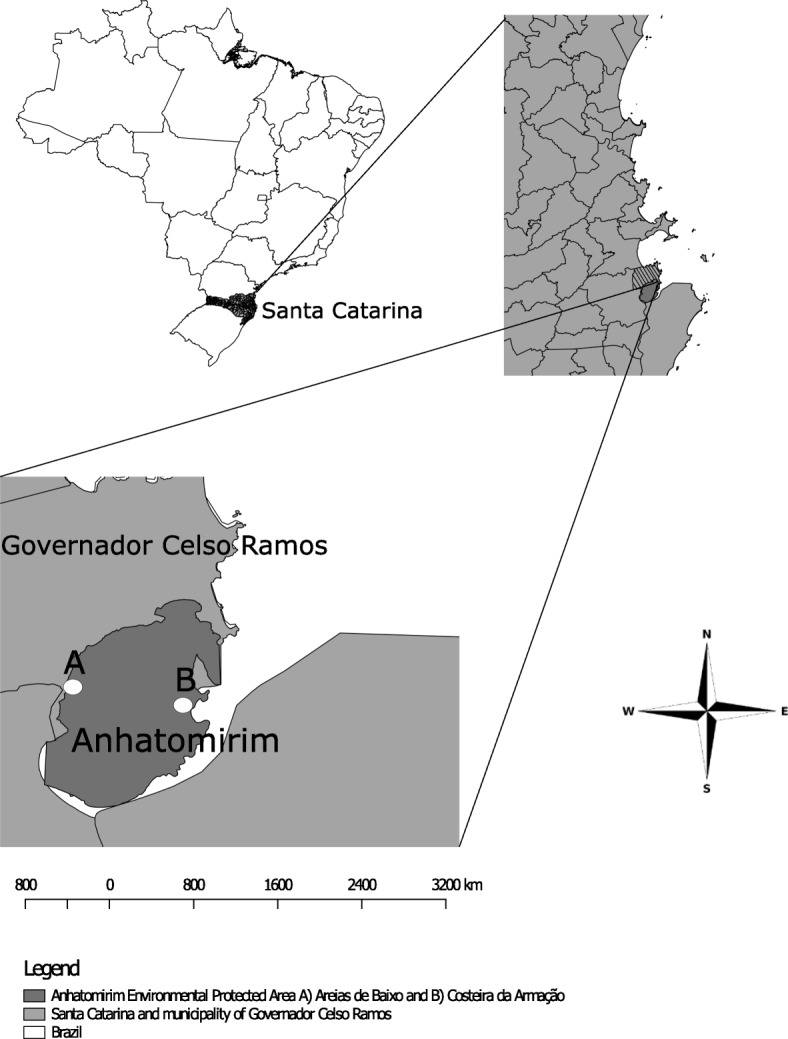
Studied communities in the Anhatomirim Environmental Protected Area, in the municipality of Governador Celso Ramos, Santa Catarina, Brazil

We conducted the study in the Anhatomirim Environmental Protected Area, in Governador Celso Ramos, Santa Catarina State, Southern Brazil. This coastal protected area was established in 1992 with the main purpose of conserving the *Sotalia guianensis* dolphin population in conjunction with the surrounding Atlantic Forest. The protected area is about 4436.56 ha and includes six communities [14], of which two were chosen for data collection: Areias de Baixo and Costeira da Armação (Fig. 1). The climate in this area is humid subtropical (Cfa), and the vegetation is ombrophilous dense forest.

These communities were chosen due to contrasting livelihoods of the inhabitants. Areias de Baixo comprises predominantly agricultural and gardening farmers who cultivate ornamental plants and crops for personal consumption. This community is partially within the protected area, and the part inside the area has about 115 inhabitants [15]. Costeira da Armação is completely within the protected area and has about 282 inhabitants [15] with a predominantly artisanal fishing economic profile. Additionally, Costeira da Armação is also known as Baía dos Golfinhos (Dolphin’s bay) due to its proximity to where dolphins (*Sotalia guianensis*) occur; thus, tourism is another important economic activity.

#### Study design, data collection, and analysis

We adopted the following criteria to include the collaborators in our sample: must be 18 years or older, a permanent resident or temporary resident for at least 5 years, and agree to participate in the research. We estimated the number of interviews according to [16]. This was based on the number of inhabitants of the communities, assumed an associated sampling error of 10% and resulted in 53 and 72 interviews for Areias de Baixo and Costeira da Armação, respectively. We conducted the interviews between November 2014 and June 2015.

We interviewed each collaborator separately. We asked about her/his socioeconomic profile, and we asked them to free list the plants they know. For each plant, we also asked about the purpose of use and we organized these purposes into categories, such as Food, Medicinal, Construction, Ornamental, and Others. Plants whose uses were cited only once or twice and for specific purposes, such as for handicrafts and superstitions, were placed in the last category (Others). We also asked about the situation of the use (present, past, or plants known but never used) and information about how they obtained the plants. We considered plants with past uses as those items used in the past, but currently not used. Plants known but never used were considered those plants which usefulness was learned about, but the interviewee did not actually use.

We collected and photographed the cited plants during walk-in-the-woods [17] in home gardens and areas of native vegetation. The plants were identified using literature [18–20] and had their names and origin checked with Tropicos [21] and Reflora [22] databases. Additionally, the first author kept a field diary to record notes with qualitative information.

We used descriptive statistics to analyze data from the interviews. Spearman correlation was tested with number of plants cited in the past and interviewees’ age. The interviews of each community were used as a proxy to reflect different livelihoods; the main economic activity in Areias de Baixo is urban jobs, while in Costeira da Armação, it is artisanal fishing. To analyze changes in the knowledge and use of plants, we used a time span corresponding to the last 55 years, approximately, and this time span was determined in relation to childhood memories of the interviewees and to changes in environmental legislation and the establishment of the protected area.

Economic activities were organized into categories: City, Agriculture and fishing, Retired, and No income. Activities developed in the City category include those carried out in urban areas, such as small commerce of clothing or food and administrative activities in public and private companies. Permanence and change in economic activities in each community were compared through a chi-squared test with a significance of 5%. For the free lists, we calculated the frequency of cited plants. To compare averages of age and number of cited plants in both communities, we used the Wilcoxon test with a significance of 5%. Qualitative analyses of the interviews and of the field diary were used to analyze driving forces for the abandonment of plant use. To represent the dynamic in permanence and change in economic activities, we used the valued directed graph *G* (*V*, *A*), where *V*={*v*∈*V* | *v* is an occupation} and *A*={(*v*_1_,*v*_2_,*n*) | *n* people that changed from occupation *v*_1_ to occupation *v*_2_}.

## Results

**Fig. 2:**
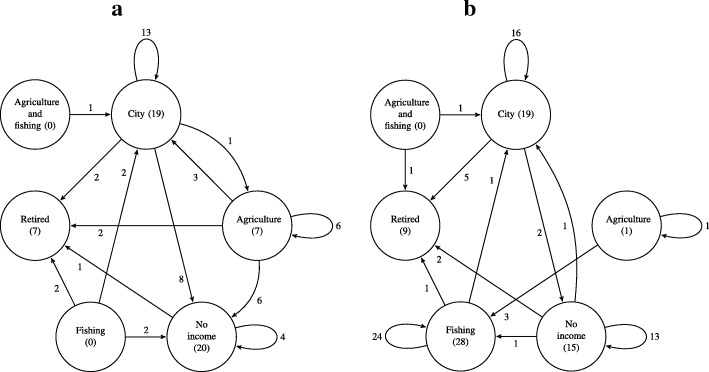
Graph of the dynamics between permanence or change in economic activities of the interviewed residents: **a** Areias de Baixo and **b** Costeira da Armação. The number indicated in parentheses represents the total number of persons currently occupying the profession indicated at the vertex. The straight arrows indicate the changes in economic activities, and the loop arrows indicate the permanence in economic activity

Most of the interviewed residents were native to the research area (Areias de Baixo, hereafter AB = 50.94%, and Costeira da Armação CA = 69.44%). The ages of the interviewees varied from 18 to 75 years old, and the average age was 48.03 years old (*σ*= 15.77) in AB and 52.62 years old (*σ*= 13.91) in CA. These averages were not significantly different (*W*= 1556, *p* value = 0.07895). Comparative data of the sampling composition can be observed in the table (Table 1). The dynamics of permanence and change of the interviewees’ economic activities (Fig. 2) show that about 13.2% of the interviewed residents from Areias de Baixo still work with agriculture, while about 36% of the interviewees from Costeira da Armação are still fishers, when compared to economic activities in the past. For permanence and change in the livelihoods, Areias de Baixo had a higher proportion of changes in economic activities compared to Costeira da Armação (*χ*^2^= 13.6017, *p* value = 0.000226).

**Table 1:** Comparative table with socioeconomic characteristics of the studied communities

	Socioeconomic characteristics	Areias de Baixo (%) (*n* = 53)	Costeira da Armação (%) (*n*= 72)
1.	Gender		
	∙ Female	58.49	45.84
	∙ Male	41.51	54.16
2.	Age		
	∙ 18–49	47.17	36.12
	∙ 50–75	52.83	63.88
3.	Economic activities in the past		
	(a) Agriculture and fishing	41.51	11.11
	(b) City	24.53	18.06
	(c) No changes in source of income	26.42	58.33
	(d) No income	7.54	12.06
4.	Economic activities in the present		
	(a) Agriculture and fishing	13.20	37.5
	(b) City	35.85	27.78
	(c) Retired	13.20	15.28
	(d) No income	37.75	19.44
5.	Residence		
	(a) Native from Governador Celso Ramos	50.94	69.44
	(b) Native from other municipalities	49.06	30.56

***Diversity of plant knowledge***

We identified 231 plant species from 97 botanical families. The most representative families, in terms of number of species, were Asteraceae (20 species), Fabaceae (19), and Lamiaceae (16). For AB, the average number of cited plants (26.81, *σ* 15.28) is higher than for CA (17.25, *σ* 12.11), and these averages are statistically different (Wilcoxon *W* = 2763.5, *p* = 1.916e −05). The number of cited plants, category of use, and number of citations for each category are shown in Table 2. In both communities, the food and medicinal categories are notable for having the most number of citations. The third most cited category was different in each community. In Areias de Baixo, it was ornamental plants, while in Costeira da Armação, it was plants for construction. The category Others (Table 2) includes plants used as handicrafts, such as *Alchornea glandulosa* Poepp. & Endl. for making wooden clogs and *Sansevieria trifasciata* Prain as protection charms for houses.

**Table 2:** Number of plants cited by category of use and number of citations for each category of use according to 53 interviewees in Areias de Baixo and 72 in Costeira da Armação

	Areias de Baixo (*n*=53)		Costeira da Armação (*n*=72)	
Category	No. of	No. of	No. of	No. of
of use	plants cited	citations	plants cited	citations
Food	97	728	98	715
Medicinal	51	299	55	280
Construction	23	85	43	208
Ornamental	61	247	20	29
Others	16	51	13	38

In both communities, the most cited plant for food was *Musa paradisiaca* L. and the most cited for medicine was *Mentha rotundifolia* (L.) Huds. As an ornamental plant, orchids (several species of Orchidaceae) was the most cited in Areias de Baixo. *Schizolobium parahyba* (Vell.) Blake was the most cited plant for the construction category in Costeira da Armação (Table 3). In Costeira da Armação, few species were cited as ornamentals, such as *Portulaca grandiflora* Hook., *Mirabilis jalapa* L., and *Euphorbia pulcherrima* Willd. ex Klotzsch, and all of them had low citation frequencies. In contrast, in Areias de Baixo, few species were cited for construction, such as *Andira anthelmia* (Vell.) J.F. Macbr., *Copaifera trapezifolia* Hayne, and *Piptadenia gonoacantha* (Mart.) J.F. Macbr, and all of them had low citation frequencies. The plants cited in both communities were strongly represented by exotic species (AB = 74.40%, CA = 65.05%) (Table 3).

**Table 3:** Most cited plants (at least 20% of the citations) for each category of use in Areias de Baixo (AB) and Costeira da Armação (CA) and citation frequency (Freq.), according to 53 interviewees in AB and 72 in CA

Category of use	Family	Species	Local name	Freq. (%) AB	Freq. (%) CA	Origin
	Rutaceae	*Citrus sinensis* (L.) Osbeck	Laranja	–	84.90%	Exotic
	Musaceae	*Musa paradisiaca* L.	Banana	71.70%	52.77%	Exotic
	Rutaceae	*Citrus* spp.	Bergamota	60.37%	–	Exotic
	Euphorbiaceae	*Manihot esculenta* Crantz	Mandioca	38.88%	60.37%	Exotic
	Myrtaceae	*Psidium guajava* L.	Goiaba	47.22%	–	Exotic
	Lamiaceae	*Mentha rotundifolia* (L.) Huds.	Hortelã	–	50.94%	Exotic
	Myrtaceae	*Plinia trunciflora* (O.Berg) Kausel	Jaboticaba	44.44%	–	Exotic
Food	Rutaceae	*Citrus* sp	Limão-de-peixe	–	56.61%	Exotic
	Myrtaceae	*Eugenia uniflora* L.	Pitanga	38.88%	–	Native
	Lamiaceae	*Mentha rotundifolia* (L.) Huds.	Hortelã	50.93%	31.94%	Exotic
	Lamiaceae	*Plectranthus barbatus* Andrews	Boldo	35.85%	33.33%	Exotic
Medicine	Amaranthaceae	*Alternanthera brasiliana* (L.) Kuntze	Pinicilina	26.41%		Native
Ornamental	Orchidaceae	Several species	Orquídea	41.51%	–	–
Construction	Fabaceae	*Schizolobium parahyba* (Vell.) Blake	Garapuvu	–	36.11%	Native

### Use, management, and shifts in plant knowledge

**Fig. 3:**
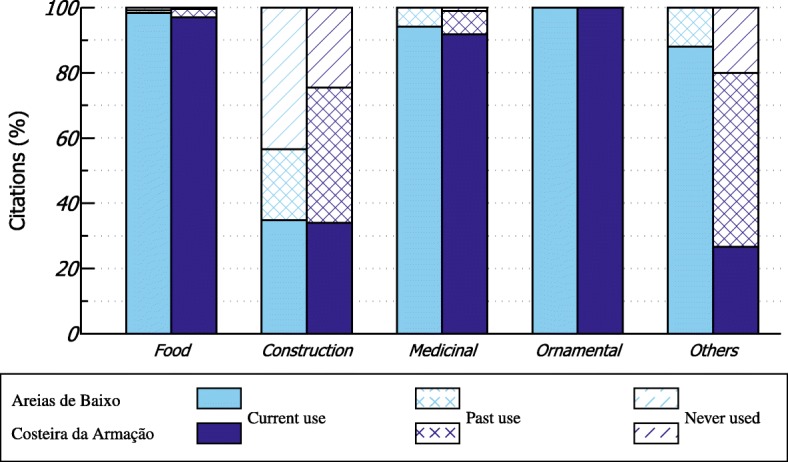
Current and past use of plants cited in the interviews with 125 residents from Areias de Baixo and Costeira da Armação

Most of the documented plants are still in use: about 90.44% in Areias de Baixo and 76.08% in the Costeira da Armação. Plants for food, medicine, and ornamental use had the most citations among the ones currently in use. In both communities, plants used in the past had 4.78% of citations in Areias de Baixo and 15.79% in Costeira da Armação. Plants cited as known but never used constituted 4.78% of the citations in Areias de Baixo and 8.13% in Costeira da Armação. We noticed a contrasting result for the construction category between the communities. In Areias de Baixo, the plants used for construction were mostly cited as known but never used, while in Costeira da Armação, they were mostly cited due to their prior use (Fig. 3).

There was no correlation between age and number of plants cited in the past in both communities (AB Spearman correlation = 0.15 and CA Spearman correlation = 0.30). When asking about the plants used in the past, we found a set of memories related to childhood and another one related to daily practices. In Areias de Baixo, plants related to childhood include *Inga* sp. as a tree used to hang swings, *Lagenaria* spp. to make toys such as rattles, and *Tanacetum vulgare* L. as a medicine used during childhood.

In Costeira da Armação, plants used in the past were mainly cited as trees used to sustain swings and tree houses (*Inga* sp. and *Hovenia dulcis* Thunb.). We also recorded one plant used as food in the past, *Momordica charantia* L., and the use of this plant was followed by the explanation that it was considered a “poor people’s food.” For past uses, the interviewees also cited plants for daily practices, such as construction, repairs, and to make utensils for daily life. For example, *Cupania vernalis* Cambess. and *Schizolobium parahyba* (Vell.) Blake were both used to build canoes, and *Piptadenia gonoacantha* (Mart.) J.F. Macbr. was used to repair boats. Other uses of plants in the daily routine in the past included soap made from *Aleurites fordii* Hemsl and cleaning artifacts such as brooms (*Baccharis* sp.).

**Fig. 4:**
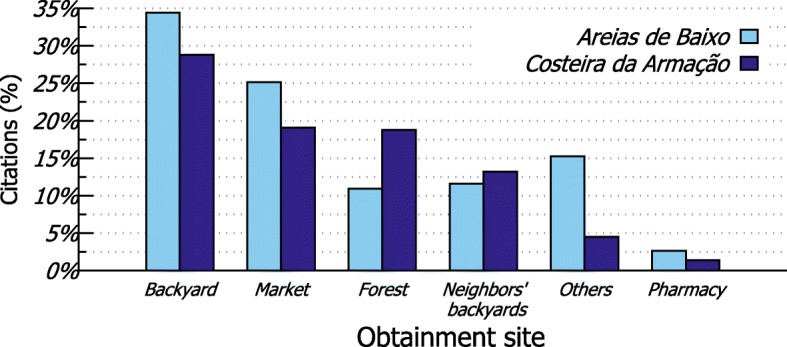
Places for obtaining plants cited in the interviews with residents from Areias de Baixo (*n*=302) and Costeira da Armação (*n*=288)

**Fig. 5:**
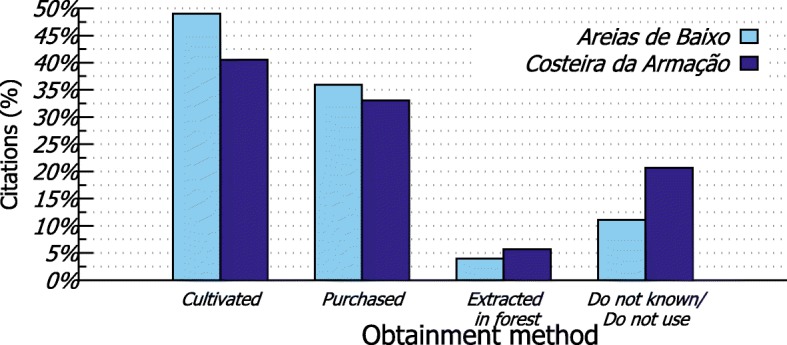
Methods for obtaining plants cited in interviews with residents from Areias de Baixo (*n*=253) and Costeira da Armação (*n*=227)

The most frequent places for obtaining plants (Fig. 4) were the following: backyards (AB = 34.44%, CA = 28.82%), markets (AB = 25.16%, CA = 19.10%), forests (CA = 18.75%), and neighbors’ backyards (AB = 11.59%, CA = 13.19%). Plants extracted from the forest (AB = 3.95%, CA = 5.73%) were mostly fruits and medicinal herbs and were used as complementary resources to what was purchased at markets (AB = 35.96%, CA = 33.04%) (Fig. 5).

## Discussion

In our research, most of the recorded plants are still in use, which indicates the close proximity of the people to plants. However, the most important environments providing these plant resources are backyards, and not the forest. In other words, even considering that they live within or very close to a protected area created to conserve Atlantic Forest, the forest itself is little used. Larios et al. [23], in a study about plant management and biodiversity conservation in Náhuatl backyards in Mexico, found that even with surrounding forest, backyards compensate for the scarcity of naturally available plant resources. Such findings make backyards keystones in strategies for conservation. In spite that backyards have received increasing attention and are considered a conservation strategy of plant diversity at the local scale [24, 25], in the present study most taxa in the set of known plants are exotic. This set of exotic plants is used for food and medicine and some of the plants are also acquired in markets and serve as a complement to what is not easily obtained from gardens or during a certain season. A higher citation of exotic plants may be related to economic factors, such as agricultural activities that are based on exotic species in Brazil [26, 27]. In addition, an explanation for the use of exotic medicinal plants could be versatility, specifically because these plants are used to treat many infirmities [28].

**Fig. 6:**
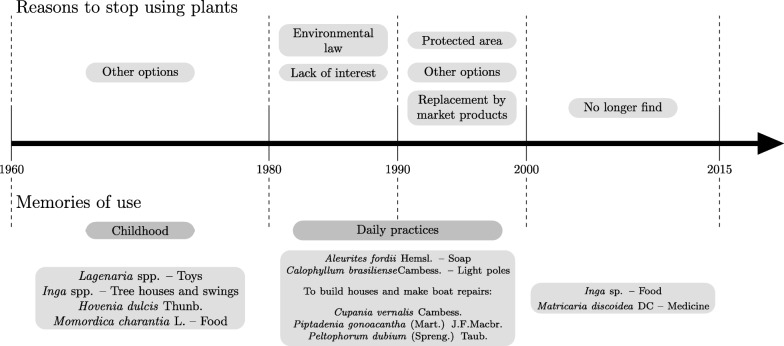
Timeline relating driving forces for the abandonment of plant use

According to the interviews, the driving forces for shifts and losses of plants used in the past were (1) better options for the same purposes or, in some cases, they (2) replaced a plant by market products; (3) loss of interest in using a plant; (4) the current environmental law, which they quoted as “too restrictive”; and (5) the fact that they no longer find some plants (Fig. 6).

Besides food and medicine, ornamental was the most cited category by residents in Areias de Baixo because this community still has gardening and agricultural economic practices. On the other hand, plants for construction were most cited in Costeira da Armação due to fishing practices that require knowledge about canoe construction. However, unlike the ornamental category, plants used for construction refer to past uses. We noted that in both communities the age of interviewees was not correlated with number of plants cited in the past. The perception of plant use in the past may be associated with a variety of factors such as environmental legislation and the management rules of the protected area. Orofino et al. [29] report a decrease in access to timber resources for building and maintaining dugout canoes by artisanal fishers in Brazilian coastal areas of central Santa Catarina State. According to their study, artisanal fishers reported difficulties in access to trees and changes in fishing activities due to current legislation that does not allow the management of native species.

Ethnobotanical studies in coastal communities show that the socioeconomic profile of these areas varies through time, attracting new residents and developing different economic activities [2, 30, 31]. There are several studies about the connection between socioeconomic changes and losses in ethnobotanical knowledge [32–35]. Although our study found a greater proportion of livelihood changes in Areias de Baixo, we noted a lower frequency of cited plants with past uses for this community. On the other hand, for Costeira da Armação, we observed a lower proportion of livelihood changes and a higher frequency of plants cited with past uses. According to [36], this can be explained because changes in economic activities do not always lead to a loss of ethnobotanical knowledge if the activities keep people in their habitat and culture.

Qualitative analysis of the field diary discussions involved evaluating potential drivers of shifts and losses of plants reported by the collaborators interviewed. We distinguished a set of memories related to childhood and day to day activities. We call attention to the “environmental law” and “protected area” drivers related to the legislation protecting the Atlantic Forest (article 225 of the Brazilian Federal Constitution [37] and the decree 99547/90 [38]) and the creation of the Anhatomirim Environmental Protected Area, which forbids the cutting of native trees without permission. Since these communities are in an environmental protected area, residents were apprehensive when talking about plants used for construction because it would involve talking about timber plants that are mostly native. Therefore, the environmental law was also mentioned in justifying the abandonment of plants used in the past. This tendency in abandonment of plants and practices was also observed in studies by [29, 30, 39].

In some cases, residents mentioned the replacement by market products, such as commercial soaps replacing those usually made of *Aleurites fordii* Hemsl. The “lack of interest to keep using a plant” was another driving factor mentioned by some residents. Once they were no longer interested in being farmers or fishers, as their parents, they gradually stopped using some plants due to personal choice. According to [3], knowledge of plants may be threatened by external factors, such as cultural and economic pressure, since it is easier to access some plant resources by purchasing them. In a study on the knowledge and management of “restinga” plants in a local community in Brazil, [2] found that the frequency of plant use decreased considerably from past to present, which can cause loss of ethnobotanical knowledge among future generations.

## Conclusion

Although the communities are close and located within the same environmental protection area, local knowledge about plants reveals particularities of the two communities. Qualitative analyses showed that while shifts in plant use seem to do more with personal choices and economic and market facilities, the loss of plant knowledge seems to be more related to drivers of environmental policies and the establishment of the protected area. We discard the hypothesis about changes in livelihoods and the reflection in plants used in the past, considering that even with changes in economic activities, ethnobotanical knowledge was embedded in the habitats and cultures of the collaborators interviewed. The maintenance of backyards can be a factor that favors ethnobotanical knowledge. Although our research has not assessed richness in backyards, the greater number of known exotic species in backyards requires further study before being used as an example for conservation strategies, especially in a protected area.

## Abbreviations

AB: Areias de Baixo
CA: Costeira da Armação
